# Hughes-Stovin Syndrome: A Rare Cause of Thrombosis and Pulmonary Artery Aneurysm

**DOI:** 10.7759/cureus.37121

**Published:** 2023-04-04

**Authors:** Selma Yalçın, Kerem Ensarioglu, Bahar Kurt, Cem Özişler

**Affiliations:** 1 Department of Pulmonology, Ordu Fatsa State Hospital, Ordu, TUR; 2 Department of Pulmonology, University of Health Sciences, Ankara Atatürk Sanatorium Training and Research Hospital, Ankara, TUR; 3 Department of Pulmonology, Ankara Etlik City Hospital, Ankara, TUR; 4 Department of Rheumatology, University of Health Sciences, Diskapi Yildirim Beyazit Training and Research Hospital, Ankara, TUR

**Keywords:** vasculitis, pulmonary embolism, pulmonary aneurysm, hughes-stovin syndrome, cyclophosphamide, behcet's disease

## Abstract

Hughes-Stovin Syndrome (HSS) is a rare clinical condition characterized by thrombophlebitis as well as multiple pulmonary and bronchial aneurysms. It commonly presents with coughing, dyspnea, fever, chest pain, and hemoptysis, and its management usually consists of surgical and medical approaches. In this report, we discuss a case of a patient with HSS. A 30-year-old male patient was admitted to the pulmonary medicine ward for hemoptysis. After evaluation with chest CT, bilateral pulmonary embolism and pulmonary aneurysms were observed. Due to a history of aphthous lesions, Behçet’s disease (BD) was considered the initial diagnosis; however, the patient did not fit the criteria and was later diagnosed with HSS. Intravenous methylprednisolone was initiated, along with a maintenance treatment with cyclophosphamide. Treatment response was observed in the fourth month; however, due to the persistence of hemoptysis, additional cycles of cyclophosphamide were later required, under which the patient's condition has been stable. HSS currently lacks clear diagnostic criteria, and further studies are needed to investigate genetic backgrounds, familial transmissions, and treatment alternatives.

## Introduction

Hughes-Stovin Syndrome (HSS) is an uncommon clinical condition, and it is characterized by thrombophlebitis, and multiple pulmonary and bronchial aneurysms [1]. Less than 40 cases of HSS have been reported in the literature so far [2]. Pathogenesis and etiology of HSS remain unknown; however, a history of infection and angiodysplasia have been reported as possible origins [3]. Some authors consider HSS to be a variant of Behçet's disease (BD) [1,3]. HSS generally presents with coughing, dyspnea, fever, chest pain, and hemoptysis. Treatment methods with surgical and medical approaches are available for HSS [3,4].

Medical treatment consists of corticosteroids and cytotoxic agents, among which cyclophosphamide is considered the primary choice [1]. Due to the possible risk of lethal hemorrhage, anticoagulation is contraindicated in HSS, even in the presence of pulmonary thromboembolism (PTE) [5,6]. However, anticoagulation may be considered in cases of massive PTE or intracardiac thrombosis [2]. Surgical resection by lobectomy or pneumonectomy may be performed in the presence of localized pulmonary aneurysms. In this report, we discuss the diagnosis and treatment of a male patient with HSS.

This case report was previously presented as a poster at the European Respiratory Society International Congress in Paris in 2018.

## Case presentation

**Figure 1:**
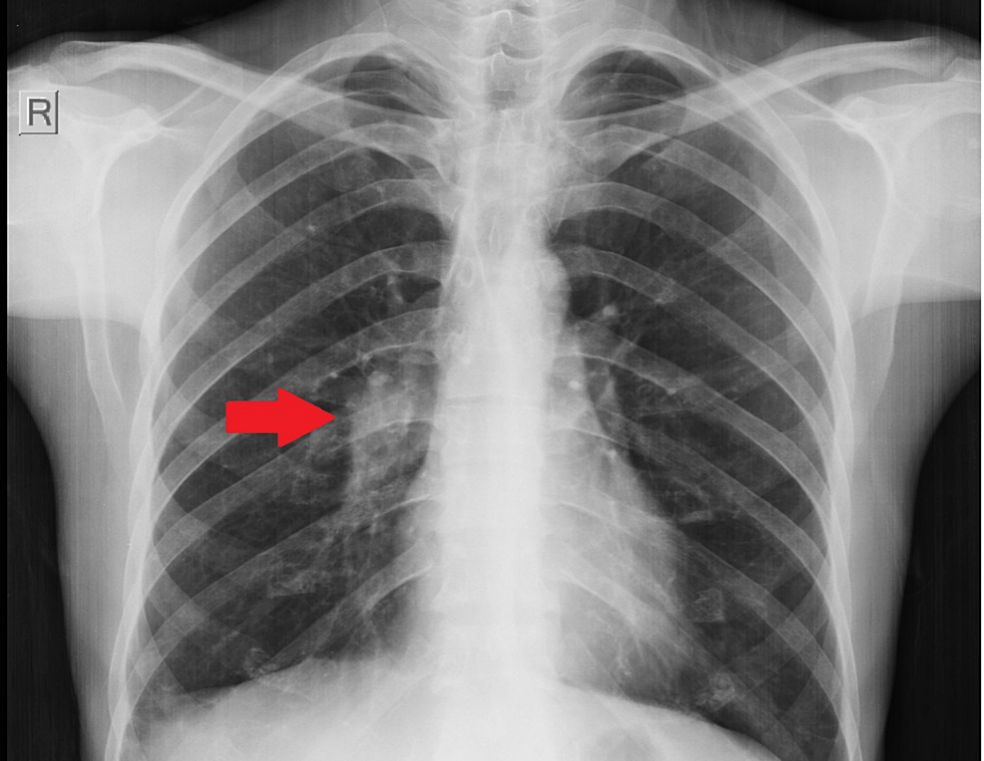
Chest X-ray on hospital admission The initial evaluation was done at the emergency ward with the chest X-ray revealing an enlarged right hilus, indicating a mediastinal or vascular pathology

**Figure 2:**
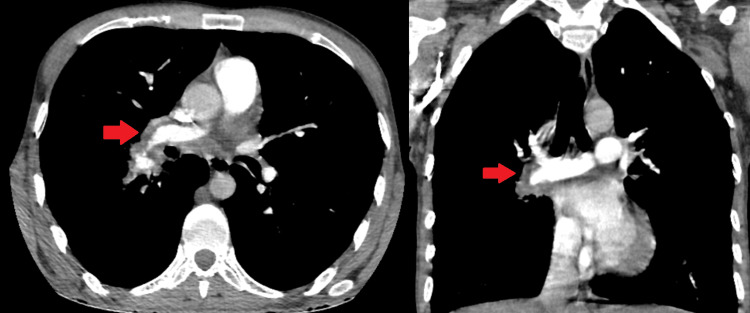
Initial evaluation with CT chest Upon admission, pulmonary thromboembolism and aneurysm in segmentary arteries were observed in the transverse and coronary sections of CT angiography of the thorax CT: computed tomography

A 30-year-old male patient presented to the emergency department (ED) with dyspnea, coughing, phlegm, fever, and night sweating for about a month; he had received antibacterial treatment at another center for these symptoms. After an initial response to the treatment, the patient presented to ED as hemoptysis began and progressively increased within a month to roughly 300 ccs per day. The initial chest X-ray revealed an enlarged right hilus and CT angiography of the thorax showed bilateral pulmonary embolism at segmentary branches of pulmonary arteries and pulmonary aneurysms (Figures 1-2).

The patient was admitted to the respiratory ward. He was hemodynamically stable and had low oxygen saturation on room air (82% with finger probe). Echocardiography, which is performed for right ventricular dilatation and strain, demonstrated normal left ventricular function with an ejection fraction of 60% and right pulmonary artery pressure <30 mmHg. As his troponin I level was below 0.05 ng/ml and he did not show any sign of cardiac decompensation, thrombolytic treatment was not initiated as the patient was not classified as high-risk. Low-molecular-weight heparin (LMWH) was initiated for PTE at a dosage of 60 mg/0.6 ml twice daily. Routine blood sampling revealed anemia, and elevated C-reactive protein (CRP, 15 mg/L, reference range: 0-5 mg/L) and erythrocyte sedimentation rate (ESR, 50 mm/h, reference range: 0-15 mm/h). To investigate the possible causes of PTE, Doppler ultrasonography was planned for the lower extremity and portal vein. Deep vein thrombosis was observed, for which the initial LMWH administration was considered adequate, along with compression stockings.

The patient reported a history of aphthous lesions in systemic evaluation, and given his young age and pulmonary aneurysm, a possible diagnosis of BD was considered. However, the patient did not fulfill the international criteria for BD, as ocular lesions, oral or genital aphthosis, skin lesions, and central nervous involvement were not present, with the only evident involvement being vascular. A diagnosis of HSS was then established after BD was ruled out, as HSS consists of deep vein thrombosis and pulmonary artery aneurysms. For the confirmation of the diagnosis and exclusion of other vasculitis pathologies, the requested rheumatologic panel was observed to be normal, which included anti-neutrophil cytoplasmic antibodies (cytoplasmic and perinuclear), anti-nuclear antibodies, and rheumatoid factor.

**Figure 3:**
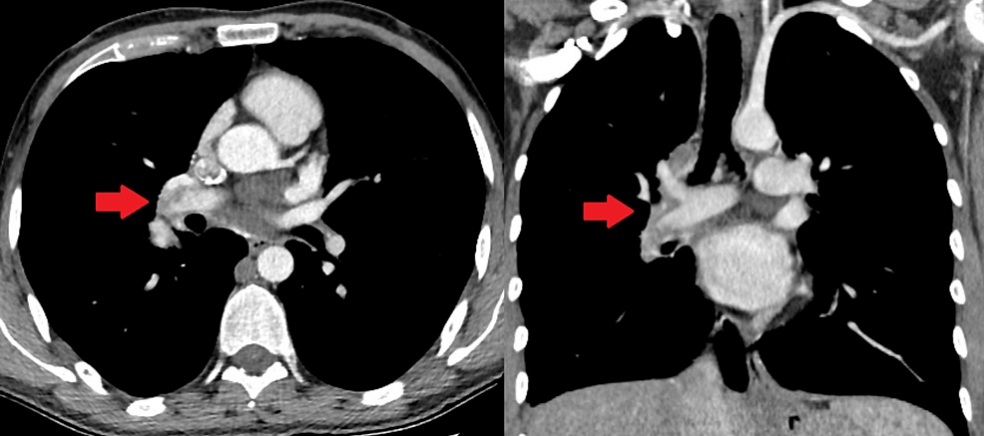
Treatment evaluation with CT chest Regression in both aneurysmatic lesions and pulmonary embolism was seen after four months of treatment CT: computed tomography

Intravenous methylprednisolone 1 gram per day was initiated for three days, followed by a 1 mg/kg/day IV schedule as the initial treatment. The steroid regimen was then tapered over a month in oral dosage. Afterward, a regimen of IV cyclophosphamide 1 gram monthly was employed. The original plan involved six cycles of cyclophosphamide, and treatment response was observed in the fourth month, with regression seen both in PTE and aneurysmatic dilatations on tomography slides (Figure 3).

**Figure 4:**
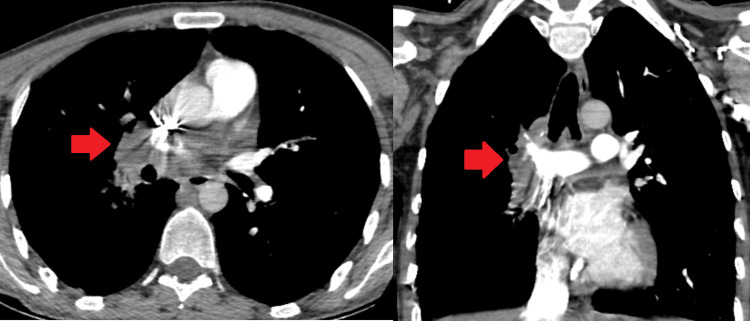
Follow-up CT chest After six months of treatment, the size of the aneurysmatic lesions increased, and hence the duration of treatment was extended CT: computed tomography

**Figure 5:**
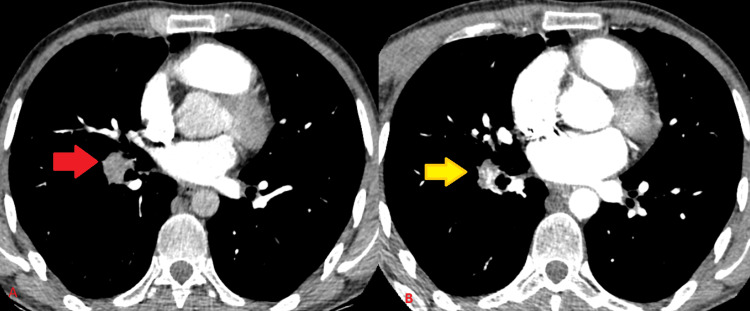
Treatment response evaluation with CT chest Compared to the sixth-month imaging (A-red arrow), reduction in pulmonary embolism and aneurysmatic structures (b-yellow arrow) led to the cessation of treatment in the ninth month CT: computed tomography

On the CT performed as part of the six-month evaluation, progression was observed and the total cyclophosphamide treatment duration was extended to nine months (Figure 4). Optimal response was observed after an additional three months of cyclophosphamide cycles (Figure 5).

After a thiopurine methyl transferase test confirmed that the level is within normal range, the patient was put on azathioprine (initial daily dosage of 50 mg) and a low-dosage methylprednisolone (16 mg initial daily dosage) regimen indefinitely, with a plan to taper off the steroid component. One year after this regimen change, the patient had to be hospitalized again due to massive hemoptysis. A reevaluation was performed in consultation with the rheumatology department, and pulse methylprednisolone 1 gram per day IV was initiated as the emergency modality. A CT with contrast at the time of reevaluation could not be performed due to elevated creatinine levels, which later responded to IV and oral hydration. A reinduction of cyclophosphamide for six months with mesna was deemed necessary, under which the patient has been stable so far.

## Discussion

The clinical paradigms of HSS can be divided into three stages [5], the first being the presence of thrombophlebitis, followed by progression to pulmonary and/or bronchial involvement, and finally, hemoptysis, which may be lethal when caused by aneurysm rupture. These stages mostly follow in order, with the first two stages considered the criteria for the diagnosis of HSS and the last stage associated with untreated patients. Most aneurysms appear in pulmonary and bronchial arteries; however, the disease is not limited to these, as shown in the study by Herb et al., which reported an HSS patient with left hepatic arterial involvement [7]. In our case, such involvement was excluded by hepatic Doppler ultrasonography.

No specific laboratory testing exists for HSS. Leukocytosis, anemia, and elevated ESR and CRP levels may be observed in laboratory evaluation. Peripheral venous thrombosis is a crucial component of HSS; thus, evaluation of possible thromboembolism with Doppler ultrasonography should be performed in patients with HSS. Conventional pulmonary angiography remains the gold standard for the diagnosis of pulmonary arterial aneurysms. Among differential diagnoses, BD is the most commonly encountered pathology with overlapping features in terms of clinical, radiologic, and histopathologic findings [1]. HSS diagnosis is thus a diagnosis of exclusion, with the presence of deep venous thrombosis and vascular involvement similar to BD, along with typical vasculitis characteristics of fever and elevated inflammatory markers, albeit the lack of expected cutaneous and nervous involvement of BD.

Immunosuppressive regimens, which include a combination of glucocorticoids and cyclophosphamide, remain the mainstay modality for the initial treatment of HSS. Steroid treatment is usually initiated as IV pulse therapy, followed by oral admission. Steroid treatment may be stopped once clinical response is observed; however, cyclophosphamide should be used for at least one year [6]. While useful in a thromboembolic situation, anticoagulation and thrombolytic agents are usually contraindicated in HSS due to the increased risk of fatal hemorrhage [4]. Surgical resection may be performed in localized aneurysms to one segment or lobe to control massive hemoptysis. This recommendation has been made in several case reports in the literature [1,4-7].

## Conclusions

HSS currently lacks clear diagnostic criteria and treatment guidelines due to its rarity, and the information currently available is limited to that found in case reports. Generally accepted diagnostic criteria and treatment algorithms need to be established to enable early diagnosis and prompt management of the disease, which may prevent morbidity and mortality. Further investigation into genetic backgrounds and familial transmissions is also required to provide possible genetic consultation for patients. HSS should be considered in the differential diagnosis of younger patients whose clinical presentation includes a combination of hemoptysis, pulmonary aneurysms, and thrombosis.
